# Clinical characteristics of psychotic disorders in patients with childhood trauma

**DOI:** 10.1097/MD.0000000000036733

**Published:** 2023-12-22

**Authors:** Sanjin Lovric, Miro Klaric, Ivona Lovric, Renata Camber, Martina Kresic Coric, Josip Kvesic, Anita Kajic-Selak

**Affiliations:** a Department of Psychiatry of the University Clinical Hospital Mostar, Mostar, Bosnia and Herzegovina; b Department of Dermatology of the University Clinical Hospital Mostar, Mostar, Bosnia and Herzegovina.

**Keywords:** domestic violence, emotional abuse, psychological abuse, psychotic disorders, suicidality

## Abstract

Childhood trauma is an important predictor of psychotic disorders, with special emphasis on physical and sexual abuse. It influences the clinical picture and course of psychotic disorders. This study was conducted in the Department of Psychiatry of the University Clinical Hospital Mostar. The sample consisted of 135 participants, aged 18 to 65 years. The screening instrument to examine cognitive status was the short version of MMSE-2. Patients’ background information was collected using a sociodemographic questionnaire constructed for this study. To determine childhood trauma, the Child Abuse Experience Inventory was used to examine physical, sexual, and emotional abuse, neglect and domestic violence. The positive and negative syndrome scale scale was used to evaluate the clinical profile of psychoticism, the SSI questionnaire was used to evaluate the severity of suicidality, and the functionality of the participants was evaluated using the WHODAS 2.0. Results indicate that a significant number of participants with psychotic disorders experienced childhood trauma, an important determinant of their illness. Participants who had witnessed abuse had more severe clinical presentations (earlier onset and longer duration of illness) and more pronounced psychotic symptomatology and a lower degree of functionality. Decreased functionality is associated with witnessing abuse and physical abuse. During the civil war, a significant percentage of the participants were in childhood and adolescent development (26.7%) and exposed to frequent emotional abuse and domestic violence. As 1 traumatic event in childhood makes a person more susceptible to more traumatic experiences during life. Childhood trauma is a serious and pervasive problem that has a significant impact on the development, course, and severity of the clinical presentation of psychotic disorders. Accordingly, it is necessary to provide continuous education to mental health workers, primarily psychiatrists, regarding childhood trauma so that treatment may be approached more systematically and a plan of therapeutic interventions may be more adequately designed, which would necessarily include psychosocial support in addition to pharmacotherapy.

## 1. Introduction

Childhood trauma represents a multilayered construct that includes physical, sexual and emotional abuse and neglect in childhood.^[1–3]^ One third of the general population experiences some form of childhood trauma during lifetime.^[4,5]^ According to research findings, traumatic events in childhood can have long-term effects on cognitive and behavioral functioning in adulthood.^[6–8]^ Present findings claim that age when a person experience childhood trauma as well as duration of traumatization, are important predictors of development of psychiatric disorders,^[3,4]^ including psychotic disorders.^[9–12]^ Moreover, childhood trauma, especially physical and sexual abuse, is an important factor in the development of psychotic disorders in adulthood.^[13–18]^ According to the literature, the risk of developing psychosis is 2 to 3 times higher in people who have experienced childhood trauma than in people who have not.^[19,20]^ In accordance with this, numerous studies have established a high frequency of childhood trauma in people with schizophrenia.^[6,21,22]^

In addition to the fact that childhood trauma is associated with an increased risk of psychotic disorder development, it is also often considered an important determinant of psychotic symptomatology in adulthood.^[3,23,24]^ According to research findings, early sexual abuse is most often associated with auditory hallucinations, while physical abuse is more commonly associated with paranoia and auditory hallucinations.^[25–27]^ Other studies link neglect in childhood with the development of persecutory delusions and negative symptomatology.^[7,22,28,29]^

In addition to increasing the risk of psychosis, it has been established that childhood trauma has an effect on clinical manifestations, the course, and the final outcome of psychotic disorders.^[4,7,30–32]^ For example, psychotic patients who experienced childhood adversities have a higher rate of suicidal thoughts and suicides compared with patients without those traumatic experiences.^[3,33]^ In addition, other studies have shown that patients with psychosis who experienced childhood trauma have lower pre-morbid functioning, earlier onset of psychosis, less effective treatment outcomes in terms of chronicity and recurrent episodes of illness, and lower rates of compliance.^[5,29,33]^

Considering the level and complexity of prolonged individual and social traumatization in war-affected Bosnia and Herzegovina,^[34]^ it is assumed that war trauma in people with psychosis will have an impact on the clinical characteristics and course of their illness. Recent studies emphasize, that childhood trauma may be related to a heightened likelihood of new trauma and can amplify the risk of poor mental health and psychotic disorders.^[35–38]^

This study aimed to determine the relationship between childhood trauma and clinical characteristics of psychotic disorders.

## 2. Participants and methods

This cross-sectional study was conducted in the Department of Psychiatry of the University Hospital Mostar in the period from January to October 2022. All patients aged 18 to 65 years who were hospitalized in the above-mentioned Department of Psychiatry due to any type of psychotic disorder (acute psychotic disorder, schizophrenia, schizoaffective disorder, unspecified psychosis or affective disorders with psychotic symptoms) were included. Diagnoses were made based on the International Classification of Diseases 10th Revision criteria by a team of experienced clinicians, psychiatrists and psychologists, with a minimum of 10 years of clinical work in the Department of Psychiatry. Psychiatrists ascertained the diagnosis by psychiatric interview and clinical picture upon admission. All patients who met the criteria for psychotic disorders by psychiatrists, during hospitalization underwent the rigorous psychological assessment that included a battery of tests (Minnesota Multiphasic Personality Inventory 201 or MMPI-2, Wartegg test, The Machover Test, Beta-II, The Mosaic Test) to evaluate and confirm diagnosis of psychotic disorder.^[39–43]^

Participants were included in the order of appearance from the day the study began, until a sample of 135 patients with psychosis was formed. All the participants were informed of the study by the principal investigator. Those who agreed to participate signed the Informed Consent form. In cases in which a participant was deemed incompetent, informed consent was signed by their legal guardian. Ethics approval was provided by Ethical Committee of Medical school of the University of Mostar (Protocol number 01-1-559/17).

After the participants signed the informed consent, the Mini Mental State Examination, short version questionnaire (Mini Mental State Examination—short version) was administered to evaluate their cognitive status, and the total score was used as the inclusion/exclusion criteria in the study. In this phase, 25 (15.6%) participants were excluded. The participants were then given a battery of tests consisting of self-assessment scales (Child Abuse Experience Inventory), SSI (scale of suicidal ideation) and WHODAS 2.0 (World Health Organization Disability Assessment Schedule) along with a sociodemographic questionnaire that they filled in the presence of the main researcher, and the PANSS (positive and negative syndrome scale) questionnaire filled out by the main researcher.

### 2.1. Instruments of the study

For this study, a sociodemographic questionnaire was constructed to collect patient background information. In addition to general sociodemographic data, the questionnaire also contained data on the onset and course of mental illness and number of previous hospitalizations.

A short version of the Mini Mental State Examination, short version questionnaire, used for clinical and research purposes, was utilized as a screening instrument to examine the cognitive status of the participants.^[44]^ This questionnaire contains 16 items where every correct answer was scored with 1 point and the total score was the sum of all the correct answers. Participants with a total score of <13 points were considered to have significant cognitive impairment and were excluded from the study.

To determine childhood trauma, the Child Abuse Experience Inventory was used to examine physical, sexual, and emotional abuse, neglect and domestic violence. The results were expressed as the sum of participants’ answers, especially for physical, emotional and sexual abuse and neglect, and at the level of the entire instrument, where a higher score indicated a higher prevalence of certain forms of abuse, which means a higher prevalence of trauma in childhood.^[45]^

The PANSS, which is widely used in research on patients with psychosis, was used to evaluate the clinical profile of psychoticism. It consists of 30 items divided into 3 subscales: positive symptoms, negative symptoms, and general psychopathology. Responses were recorded on a Likert scale from 1 to 7, where a higher number indicated more pronounced symptomatology.^[46]^

The SSI questionnaire was used to evaluate the severity of the suicidality. It is a semi-structured interview that evaluates 3 dimensions of suicidal ideation: active suicidal desire, the existence of plans to commit suicide, and passive suicidal desire. The higher the total score, the more serious is the suicidal ideation.^[47]^

The functionality of the participants was evaluated using the WHODAS 2.0. This questionnaire assessed the level of functionality in 6 areas: understanding and communication, mobility, self-care, interacting with other people, life activities, and participation in community activities. Higher scores indicated a higher level of disability in each examined area and at the level of the entire instrument.^[48]^

### 2.2. Statistical analysis

Statistical analysis was performed using SPSS software (version 25.0; IBM, Armonk, NY). Descriptive statistics were applied to analyze social demographic data and results on Child Abuse Experience Inventory. The continuous data were described as the mean ± SD. The categorical data were described as N (%). A Spearmans correlation analysis was performed to evaluate the independent correlation between different types of childhood abuse (for non-normal distributed data determined using Kolmogorov-Smirnov test).

Hierarchical multiple regression analysis was conducted to explore the association between traumatization and functionality and psychotic symptomatology. Potential predictors with high co-variability (*R* > 0.7) were excluded from the model to reduce over-parameterization (psychological abuse).

The variance inflation factor was <10, which suggested there was no collinearity between variables. The contribution of predictor variables regarding childhood trauma to the explanation of psychotic disorders onset, was verified using ordinal regression analysis. The probability level of *P* < .05 was taken as statistically significant.^[49]^

## 3. Results

### 3.1. Sociodemographic data

This study included 135 participants who were diagnosed with a psychotic disorder, among whom 80 (59.1%) were male, and 55 (42.7%) were female. The average age of the participants was 37 years (SD = 11.94). The majority, (93.3%), grew up in a complete family, and 6.7% lived in an incomplete family. The other sociodemographic data and basic descriptive parameters of the Child Abuse Experience Inventory (where higher results represented greater exposure to abuse throughout childhood) are summarized in Table 1.

**Table 1:** Baseline characteristics of study participants and basic descriptive parameters of results on Child Abuse Experience Inventory.

		Total N = 135
Marital status	Single	102 (75.6%)
	Married	28 (20.7%)
	Divorced	2 (1.5%)
	Widow/er	3 (2.2%)
Children	Yes	32 (23.7%)
	No	103 (76.3%)
Work status	Employed	39 (28.9%)
	Unemployed	96 (71.1%)
Monthly income	Yes	73 (54.1%)
	No	62 (45.9%)
Child Abuse Experience Inventory	Psychological abuse	12.985 ± 8.312
	Physical abuse	3.356 ± 4.002
	Sexual abuse	3.237 ± 5.603
	Neglect	3.341 ± 3.716
	Witnessing abuse	16.948 ± 12.943
	Abuse (total result)	39.867 ± 27.669

Data are expressed as mean and standard deviation. Qualitative variables are presented as frequency and percentage.

A significant positive correlation was established between different types of abuse (r_s_ = 0.396–0.905, *P* < ..001): participants who were more often exposed to 1 type of abuse during childhood, were more often exposed to other types of abuse, or had witnessed abuse more often.

### 3.2. Impact of childhood trauma and participation in war, exile and divorce of parents on the onset of psychotic disorders

The contribution of predictor variables regarding childhood trauma, sociodemographic variables (age and divorce of parents), participation in war and exile to the explanation of psychotic disorder onset, were verified using an ordinal regression analysis (Table 2). The selected set of predictors explained approximately 32.6% (r2 Naglkerkea) of the variance in the results related to the onset of psychotic disorders, of which age and witnessing abuse significantly contributed to the explanation of the criterion variable. Participants who are now older, as expected, had earlier onset of psychotic disorder and longer duration of illness, as well as those who witnesses abuse in childhood.

**Table 2:** Results of ordinal logistic regression analysis for onset of psychotic disorder.

		Estimates	S.E.	Wald	OR	*P*
Onset	last 5 years	4.389	0.877	25.050	80.594	.000
	last 10 years	5.171	0.914	32.020	176.161	.000
	last 15 years	6.094	0.966	32.020	443.346	.000
Child Abuse Experience Inventory	Physical abuse	0.046	0.075	0.374	1.047	.541
	Neglect	−0.069	0.084	0.666	0.934	.414
	Sexual abuse	0.003	0.041	0.004	1.003	.948
	Witnessing abuse	0.044	0.020	4.746	1.045	.029*
Participation in war	Active	0.931	0.652	2.037	2.537	.153
	Passive	1.133	0.613	3.415	3.104	.065
Divorce of parents	Yes	−0.666	0.983	0.459	0.514	.466
Exile	Only me	0.056	0.846	0.004	1.058	.947
	Only family	−1.716	0.982	3.052	0.180	.081
	Family and me	−0.627	0.548	1.313	0.534	.243
	Age	0.080	0.020	16.723	1.084	.000**

* *P* < .05

** *P* < .001.

### 3.3. Impact of childhood trauma and participation in war, exile and divorce of parents on functionality of psychotic patients

Table 3 displays the results of the hierarchical multiple regression analysis where childhood abuse (Model 1) was significant and accounted for 7.9% of the variance in the WHODAS results. In Model 2, inserting participation in war, exile and divorce of parents led to an increase of 2.5% in the variance explained compared to Step 1 (for a total of 10.4%). In this analysis, witnessing abuse and physical abuse were found to be significant influencing factors. A higher level of witnessing abuse in childhood and a lower level of physical abuse are associated with a higher result on the WHODAS questionnaire which indicates lower functionality of psychotic patients or higher levels of disability in psychotic patients.

**Table 3:** Hierarchical multiple regression analysis results for level of functionality psychotic patients expressed through the result on the WHODAS 2.0.

	Model 1	Model 2
Physical abuse	−0.177	−0.253*
Neglect	0.116	0.153
Witnessing abuse	0.206	0.231*
Sexual abuse	0.117	0.119
Participation in war		−0.109
Exile		−0.083
Divorce of parents		0.053
F	2.779*	2.108*
Adjusted R^2^	0.050	0.055
R^2^	0.079	0.104

Model 1 included physical abuse, neglect, witnessing abuse.

Model 2 added participation in war, exile and divorce of parents.

* *P* < .05.

### 3.4. Impact of childhood traumatization, participation in war, age, exile and divorce of parents on severity of clinical presentation psychotic disorders

Table 4 displays the results of the hierarchical multiple regression analysis where childhood abuse (Model 1) accounted for 8.1% of the variance in the PANSS results. In Model 2, inserting participation in war, age, exile and divorce of parents led to an increase of 13% in the variance explained compared to Step 1 (for a total of 21.1%). Age and witnessing abuse were significant influencing factors—participants who were younger and witnessed abuse had more pronounced psychotic symptomatology.

**Table 4:** Hierarchical multiple regression analysis results for psychotic symptomatology expressed through the result on PANSS.

	Model 1	Model 2
Physical abuse	−0.028	−0.075
Neglect	0.010	0.013
Witnessing abuse	0.203	0.212*
Sexual abuse	0.158	0.087
Participation in war		0.087
Exile		−0.152
Divorce of parents		0.009
Age		−0.314**
F	2.875*	4.211**
Adjusted R2	0.053	0.161
R2	0.081	0.211

Model 1 included physical abuse, neglect, witnessing abuse.

Model 2 added participation in war, exile and divorce of parents.

* *P* < .05.

** *P* < .001.

PANSS = positive and negative syndrome scale.

The Impact of childhood trauma, age, participation in war, exile and parental divorce on suicidal ideation (SSI) was determined using hierarchical multiple regression analysis and it was found that the overall model was not statistically significant (R^2^ = 0.091, F = 1.387, df = 9, *P* = .201).

### 3.5. Association between childhood trauma, functionality and clinical presentation of psychotic disorder

Examining the relationship between the incidence and type of childhood trauma with severity of clinical presentation shown through the results of the PANSS questionnaire using Spearman correlation, demonstrated that there is a positive and weak association between general psychopathology on PANSS and all types of childhood traumatization (r_s_ = 0.188 (*P* < .05)–0.312 (*P* < .001)), as well as between positive symptoms and sexual abuse (r_s_ = 0.195, *P* < .05), negative symptoms and sexual abuse (r_s_ = 0.190, *P* < .05) and neglect (r_s_ = 0.177, *P* < .05). A week association was found between suicidal ideation and neglect, witnessing abuse and abuse (total score), and between neglect (r_s_ = 0.223, *P* < .001), psychological abuse (r_s_ = 0.195, *P* < .05), witnessing abuse (r_s_ = 0.236, *P* .2.001) and total score on WHODAS (level of functionality). These results are presented in Supplemental Digital Content (Tables 2–4, http://links.lww.com/MD/L118, http://links.lww.com/MD/L119, http://links.lww.com/MD/L120).

## 4. Discussion

The results of this study indicate that a significant number of people with psychotic disorders experienced some form of childhood trauma, and that childhood trauma is a significant determinant of their illness. Individuals with psychotic disorders who had experienced childhood trauma and participated in war had more severe clinical presentations and lower degrees of functionality. These findings are in agreement with other studies that have shown that childhood trauma is an important predictor of worse treatment outcomes, lower compliance rates and reduced social functioning in patients with psychotic and non-psychotic mental disorders.^[3,5,9,30,50,51]^ The same can be connected with the high degree of individual and systemic traumatization that the parents of the participants experienced during the 5-year period of the civil war that was held in this area 3 decades ago.^[34]^

It should be noted that a significant percentage of the participants in this study, during the period of war, were in childhood and adolescent development (26.7%) and were presumably exposed to frequent emotional abuse and often witnessed domestic violence. Previous studies are clear in their findings that dysfunctionality is more often present in war traumatized families, as well as all forms of family violence.^[52,53]^ Young people who went to war, escaping from such unfavorable family and social systems, and having experienced a series of traumatic experiences in the war, increased the risk of developing psychotic disorders according to the principle of the cumulative effect of trauma.^[3,54,55]^

The severity of clinical presentation assessed using the PANSS questionnaire indicated that the age and witnessing abuse are significant influencing factor in emergence of psychotic disorder: younger participants and those who witnessed abuse had more pronounced psychotic symptomatology. The feeling of constant anticipation, mistrust and unfavorable growing conditions in war-traumatized families obviously led to serious mental damage and contributed to the development of productive symptomatology of psychotic disorders.^[56–58]^ Trauma in childhood of high intensity, makes a person in adulthood more susceptible to more intense responses to stress and the development of psychological distress.^[3,20,59]^ A significant positive correlation between different types of abuse was found in this study, which means that participants who were more often exposed to 1 type of abuse in childhood were more often exposed to other types of abuse or had witnessed abuse more often (This result is presented in Supplemental Digital Content, Table 1, http://links.lww.com/MD/L117). Moreover, traumatogenic neurodevelopmental model is based on this concept.^[3]^ The weak association between childhood traumatization and clinical representation of psychotic disorders indicates the existence of certain associations, but also the need for further examination of the characteristics of that association and the need to consider other relevant factors that can influence the severity of the clinical picture of psychotic disorders.

The clinical course of illness was evaluated based on the suicidality and degree of functionality. Decreased functionality in individuals with psychosis is associated with witnessing abuse. This finding could be expected considering that adverse childhood experiences are related to the severity of clinical course of illness and level of functionality in everyday life.^[3,23,53,60–63]^ Furthermore, other studies have pointed out that active or passive participation in war was found to have a significantly negative effect on mental health and overall functioning.^[37,64]^ Given that relationships in early childhood are key to the establishment of healthy interpersonal relationships throughout life, disturbed harmony of relationships in primary families results in difficulties in later interpersonal relationships.^[22,30,65]^ We should certainly not ignore the impaired family support in treatment and life, which apparently war-traumatized and dysfunctional families were not able to provide their members with mental disorders.^[34,66]^ This finding is consistent with the results of other studies’, which found that patients who lack social support will have poorer disease control in the form of irregularly taking the prescribed therapy and irregularly going to the psychiatrist checkups.^[57,63,67]^ Childhood trauma represents an additional burden on top of the already existing series of unfavorable life factors, such as reduced social network, lack of social support, lack of functionality in everyday activities, a more severe clinical presentation, the existence of psychiatric comorbidities, unemployment and a single lifestyle.

This study had several limitations, the most important of which were the size and structure of the sample. As emphasized earlier, the sample is convenient and, as such, is certainly a less ideal choice than any probabilistic sample. Bonferroni correction was not performed for the comparisons in our study. There is, however, some debate in this regard, as it can increase type II error, cause significant differences to be considered insignificant, reduce statistical power, and cause bias. Therefore, publications are suggesting that Bonferroni correction should not be done, and it is not conducted in this study.^[68]^

The metric limitation is the nonexistence of a single criterion for determining the incidence of experienced abuse, that is, the determination of a threshold score, that does not enable the exact degree of exposure to traumatic events in an individual. Therefore, it is not possible to determine the exact number of participants with childhood trauma, but only the frequency of its occurrence. The study was conducted transversally and the most of the collected data were based on the participants’ self-reporting.

It is important to note that that the main focus of this study was to examine the relationship between childhood trauma and psychotic disorders, and bivariate correlations, among other analysis, showed significant, but weak correlations among the examined variables (presented in Supplemental Digital Content). As this approach showed a possible correlation between examined variables it is worth mentioning that it is necessary for future studies to try to identify the exact variable that has the most impact on developing psychotic disorders in order to define more precisely the relationship between traumatic experiences and the onset of psychosis.

We conclude that childhood trauma is a serious and pervasive problem that has a significant impact on the development, course, and severity of the clinical presentation of psychotic disorders. The importance of childhood trauma directly influences a more comprehensive understanding of the mechanism of psychosis development and contributes to an understanding of the social aspect of the emergence of psychotic disorders. Accordingly, it is necessary to provide continuous education to mental health workers, primarily psychiatrists, regarding childhood trauma, so that treatment may be approached more systematically and a plan of therapeutic interventions may be more adequately designed, which would necessarily include psychosocial support in addition to pharmacotherapy.

## Acknowledgments

We would like to thank Megan Perlleshi for her help in providing her with English knowledge, so this article might sound more natively English.

## Author contributions

**Conceptualization:** Sanjin Lovric, Miro Klaric.

**Data curation:** Ivona Lovric.

**Formal analysis:** Anita Kajic-Selak.

**Investigation:** Renata Camber, Martina Kresic Coric, Josip Kvesic.

**Methodology:** Sanjin Lovric, Miro Klaric, Anita Kajic-Selak.

**Supervision:** Sanjin Lovric, Miro Klaric.

**Visualization:** Sanjin Lovric.

**Writing – original draft:** Sanjin Lovric.

**Writing – review & editing:** Sanjin Lovric.

## Supplementary Material








