# Supplementary material for: Clinical characteristics of psychotic disorders in patients with childhood trauma
Source: Medicine (Baltimore). 2023 Dec 22;102(51):e36733. doi: 10.1097/MD.0000000000036733 (PMC10735130; doi:10.1097/MD.0000000000036733)
Supplement: Supplementary file 1 [file medi-102-e36733-s001.docx]

**SUPPLEMENTAL DIGITAL CONTENT**

**(Tables)**

**Table 2.** Correlation between childhood trauma and severity of clinical presentation presented through results on the PANSS questionnaire (*N=135*)

| **Type of abuse** | **PANSS**  **(Total score)** | **PANSS**  **(Positive symptoms)** | **PANSS**  **(Negative symptoms)** | **PANSS**  **(General psychopathology)** |
| --- | --- | --- | --- | --- |
| Physical abuse | .188* | .104 | .035 | .209* |
| Psychological abuse | .262* | .138 | .122 | .261** |
| Neglect | .219* | .099 | .177* | .204* |
| Witnessing abuse | .282** | .143 | .161 | .273** |
| Sexual abuse | .312** | .195* | .190* | .277** |
| Abuse (total score) | .310** | .161 | .181* | .299** |

*Spearman correlation analysis, **P<.001* P<.05*
