# Supplementary material for: Clinical characteristics of psychotic disorders in patients with childhood trauma
Source: Medicine (Baltimore). 2023 Dec 22;102(51):e36733. doi: 10.1097/MD.0000000000036733 (PMC10735130; doi:10.1097/MD.0000000000036733)
Supplement: Supplementary file 2 [file medi-102-e36733-s002.docx]

**SUPPLEMENTAL DIGITAL CONTENT**

**(Tables)**

**Table 3.** Correlation of the level of suicidality (SSI) and suicide attempts with childhood trauma (*N=135*)

| **Type of abuse** | **SSI** | **Suicide attempts** |
| --- | --- | --- |
| Physical abuse | .164 | -.139 |
| Psychological abuse | .135 | -.154 |
| Neglect | .241** | -.126 |
| Witnessing abuse | .256* | -.267** |
| Sexual abuse | .0084 | -.113 |
| Abuse (total score) | .245** | -.254** |

*Spearman correlation analysis, **P<.001* P<.05*
