# Supplementary material for: Clinical characteristics of psychotic disorders in patients with childhood trauma
Source: Medicine (Baltimore). 2023 Dec 22;102(51):e36733. doi: 10.1097/MD.0000000000036733 (PMC10735130; doi:10.1097/MD.0000000000036733)
Supplement: Supplementary file 3 [file medi-102-e36733-s003.docx]

**SUPPLEMENTAL DIGITAL CONTENT**

**(Tables)**

**Table 4.** Relationship between the type of abuse and the level of functionality of participants expressed through the results on WHODAS 2.0 questionnaire (*N=135*)

|  | **WHODAS**  **(total score)** | **Understanding and communication** | **Mobility** | **Self-care** | **Interaction with people** | **Life activities** | **Participation in community activities** |
| --- | --- | --- | --- | --- | --- | --- | --- |
| 1. Physical abuse | 0.038 | -0.008 | 0.085 | 0.051 | 0.078 | -0.09 | 0.050 |
| 2. Psychological abuse | 0.195* | 0.164 | 0.159 | 0.162 | 0.241** | 0.113 | .0167 |
| 3. Neglect | 0.223** | 0.218* | 0.220* | 0.264** | 0.199* | 0.193* | 0.139 |
| 4. Witnessing abuse | 0.236** | 0.177* | 0.202* | 0.284** | 0.202* | 0.192* | 0.195* |
| 5. Sexual abuse | 0.113 | .106 | .039 | .090 | .082 | 0.062 | 0.141 |
| 6. Abuse (total score) | 0.236** | .190* | .191* | .244** | .234** | 0.169* | 0.203* |

*Spearman correlation analysis, **P<.001* P<.05*
