# Supplementary material for: Clinical characteristics of psychotic disorders in patients with childhood trauma
Source: Medicine (Baltimore). 2023 Dec 22;102(51):e36733. doi: 10.1097/MD.0000000000036733 (PMC10735130; doi:10.1097/MD.0000000000036733)
Supplement: Supplementary file 4 [file medi-102-e36733-s004.docx]

**SUPPLEMENTAL DIGITAL CONTENT**

**(Tables)**

**Table 1.** Matrix of intercorrelations between results on the Child Abuse Experience Inventory *(N=135)*

|  | **1.** | **2.** | **3.** | **4.** | **5.** | **6.** |
| --- | --- | --- | --- | --- | --- | --- |
| **1.** Physical abuse | - | .658** | .527** | .745** | .606** | .751** |
| **2.** Psychological abuse |  | - | .607** | .684** | .462** | .863** |
| **3.** Neglect |  |  | - | .707** | .396** | .787** |
| **4.** Witnessing abuse |  |  |  | - | .419** | .905** |
| **5.** Sexual abuse |  |  |  |  | - | .600** |
| **6.** Abuse (total result) |  |  |  |  |  | - |

Spearman correlation analysis, ***P*<.001* *P*<.05
